# Fractured morphology of femoral head associated with subsequent femoral neck fracture: Injury analyses of 2D and 3D models of femoral head fractures with computed tomography

**DOI:** 10.3389/fbioe.2023.1115639

**Published:** 2023-01-17

**Authors:** Shenghui Wu, Wei Wang, Ruiyang Li, Jingyi Guo, Yu Miao, Guangyi Li, Jiong Mei

**Affiliations:** ^1^ Department of Orthopedic Surgery, Shanghai Sixth People’s Hospital Affiliated to Shanghai Jiao Tong University School of Medicine, Shanghai, China; ^2^ Department of Biomedical Engineering, The Hong Kong Polytechnic University, Hong Kong, China; ^3^ Clinical Research Center, Shanghai Sixth People’s Hospital Affiliated to Shanghai Jiao Tong University School of Medicine, Shanghai, China

**Keywords:** femoral head fracture, femoral neck fracture, 2D, 3D, injury model, computed tomography

## Abstract

**Background:** The injury of femoral head varies among femoral head fractures (FHFs). In addition, the injury degree of the femoral head is a significant predictor of femoral neck fracture (FNF) incidence in patients with FHFs. However, the exact measurement methods have yet been clearly defined based on injury models of FHFs. This study aimed to design a new measurement for the injury degree of the femoral head on 2D and 3D models with computed tomography (CT) images and investigate its association with FHFs with FNF.

**Methods:** A consecutive series of 209 patients with FHFs was assessed regarding patient characteristics, CT images, and rate of FNF. New parameters for injury degree of femoral head, including percentage of maximum defect length (PMDL) in the 2D CT model and percentage of fracture area (PFA) in the 3D CT-reconstruction model, were respectively measured. Four 2D parameters included PMDLs in the coronal, cross-sectional and sagittal plane and average PMDL across all three planes. Reliability tests for all parameters were evaluated in 100 randomly selected patients. The PMDL with better reliability and areas under curves (AUCs) was finally defined as the 2D parameter. Factors associated with FNF were determined by binary logistic regression analysis. The sensitivity, specificity, likelihood ratios, and positive and negative predictive values for different cut-off values of the 2D and 3D parameters were employed to test the diagnostic accuracy for FNF prediction.

**Results:** Intra- and inter-class coefficients for all parameters were ≥0.887. AUCs of all parameters ranged from 0.719 to 0.929 (*p* < 0.05). The average PMDL across all three planes was defined as the 2D parameter. The results of logistic regression analysis showed that average PMDL across all three planes and PFA were the significant predictors of FNF (*p* < 0.05). The cutoff values of the average PMDL across all three planes and PFA were 91.65% and 29.68%. The sensitivity, specificity, positive likelihood ratio, negative likelihood ratio, predictive positive value and negative predictive value of 2D (3D) parameters were 91.7% (83.3%), 93.4% (58.4%), 13.8 (2.0), 0.09 (0.29), 45.83% (10.87%), and 99.46% (98.29%).

**Conclusion:** The new measurement on 2D and 3D injury models with CT has been established to assess the fracture risk of femoral neck in patients with FHFs in the clinic practice. 2D and 3D parameters in FHFs were a feasible adjunctive diagnostic tool in identifying FNFs. In addition, this finding might also provide a theoretic basis for the investigation of the convenient digital-model in complex injury analysis.

## Background

Femoral head fractures (FHFs) are usually associated with high-energy trauma and posterior hip dislocation. The major choice of surgical FHF treatment was reduction and internal fixation, or fragment removal, while joint replacement surgeries were favored for some FHF patients due to the presence of the ipsilateral femoral neck fracture (FNF) (Menger et al., 2021). The ipsilateral FNF had an incidence of 8.6% and the worst prognosis among all FHFs (Menger et al., 2021 Giannoudis et al., 2009). There were many possible scenarios, simultaneously with FHF by trauma, during reduction, during an eventual FHF fixation, upon resumption of the weight bearing, *etc.*, for the occurrence of the ipsilateral FNF.

In the Pipkin classification, a single FHF was called Pipkin I or II, and associated fractures of the femoral neck and acetabulum were respectively defined as Pipkin III and Ⅳ (Pipkin, 1957). During treatment, the ipsilateral FNF is a severe intraoperative and postoperative complication of closed reduction and internal fixation of FHF with posterior hip dislocation. This may turn Pipkin I and II into a rare Pipkin III, with increased risk of femoral head avascular necrosis (AVN) (Park et al., 2016). Open reduction is the most commonly used method for iatrogenic FNF prevention in clinical practice. However, the ability of open reduction to reduce incidence of AVN is controversial. Although open reduction can lower the risk of intraoperative FNF caused by closed reduction, it is associated with complications of AVN, with a high risk of injury to vascular supply to the femoral head (Guo et al., 2010). Moreover, open reduction may fail to reverse AVN followed by joint replacement, when femoral neck refractures occur after FHF internal fixation without any injury. Hence, early recognition of the risk of FNF in FHFs is crucial, as the early identified characteristics of this injury type could help to make a more rational treatment strategy and improve the prognosis. However, there is a lack of prediction tools in clinical practice.

Previous studies have reported an association between the injury degree of femoral head and the incidence of FNF in FHFs (Davis, 1950; Henle et al., 2017). FHF is generally characterized by development of a shearing force against the acetabular rim caused by injury to the hip joint (Davis, 1950; Thompson and Epstein, 1951). The contact area between the femoral head surface and acetabular wall surface determines whether FHFs occur with or without the additional osseous lesion on the femoral neck (Henle et al., 2017). When the contact area of the femoral head surface is larger, more of the axial compression force transmitted to the hip is distributed to the surrounding bone. This results in FNFs besides FHFs. Hence, the injury degree of femoral head is a significant predictor of FNF incidence in patients with FHFs (Davis, 1950; Thompson and Epstein, 1951; Henle et al., 2017).

To date, no study has described an accurate and reliable method for measuring the injury degree of femoral head for predicting the incidence of FNF in FHFs. In addition, the hip geometry for predicting diseases of proximal femur has been studied extensively, but the exact measurement regarding FHFs has yet been clearly defined (Kazemi et al., 2016; Wells et al., 2017; Hesper et al., 2018). Moreover, clinically suspected FHF is routinely assessed using computed tomography (CT) examinations over the last few decades. Comprehensive analysis of two-dimensional (2D) and three-dimensional (3D) human models based on CT revealed femoral morphology in hip diseases (Wells et al., 2017; Hesper et al., 2018). Therefore, it is now possible to design a digital tool for identifying the risk of FNF in FHFs based on CT imaging.

This study aimed to design a method for measuring the injury degree of femoral head based on 2D and 3D injury models with CT. The secondary aim was to investigate the association of the injury degree of femoral head with incidence of FNFs in FHFs with CT. We hypothesized that 2D and 3D CT-based parameters were reliable predictors of FNF in patients with FHFs.

## Methods

### Study design

This study was approved by the Institutional Review Board (IRB) of our institution [No.2022-KY-026(K)] and followed the Strengthening the Reporting of Observational Studies in Epidemiology (STROBE) reporting guidelines for cross-sectional studies (von Elm et al., 2007). Given the retrospective nature of this study, participant informed consent was waived by IRB.

### Participants

Using a prospectively maintained orthopedic database at a large level-I trauma center, we retrospectively analyzed CT images of the hip joint in patients diagnosed with FHF between 2011 and 2022. Three investigators independently reviewed the imaging data of all FHFs to identify initially missed FHFs. A total of 228 FHFs in 228 patients were included. Patients with insufficient or poor quality axial CT images (i.e., images with severe artifacts) (*N* = 12), unclosed epiphyseal line of the femoral head (*N* = 2), pathological fracture (*N* = 3), or skeletal immaturity (*N* = 2) were also excluded. Finally, 209 hips in 209 patients were included, and there were 12 cases with FHF and FNF. Patient characteristics and features of FHFs are summarized in Table 1.

**TABLE 1:** Background characteristics of patients with femoral head fractures.

Characteristics	
Side of injury [no. (%)]	
Left	102 (48.8%)
Right	107 (51.2%)
Age (yr)	39.85 ± 16.41
Sex [no. (%)]	
Male	169 (80.9%)
Female	40 (19.1%)
Pipkin Classification [no. (%)]	
I	3 (1.4%)
II	60 (28.7%)
III	8 (3.8%)
IV	138 (66.0%)
2D Measurement-Percentage of maximum defect length (%)	
Coronal plane	76.41 ± 15.52
Cross-sectional plane	78.82 ± 16.45
Sagittal plane	78.57 ± 17.10
Average	77.93 ± 13.51
3D Measurement-Percentage of fracture area (%)	26.76 ± 12.95

### Injury model generation

Raw CT data of patients with femoral head fractures were obtained and imported into the Mimics software (Materialise, Leuven, Belgium) for image analysis. The 2D injury model of FHF was defined as an evident 2D image of maximum bone defect of femoral head in each CT plane. The 3D injury model of FHF was defined as the 3D-reconstruction model of the 3D-CT data using 3-matic software (Materialise, Belgium). Additional details of the reconstruction of 3D model analysis are given in the Appendix A.

### Measurements

**FIGURE 1:**
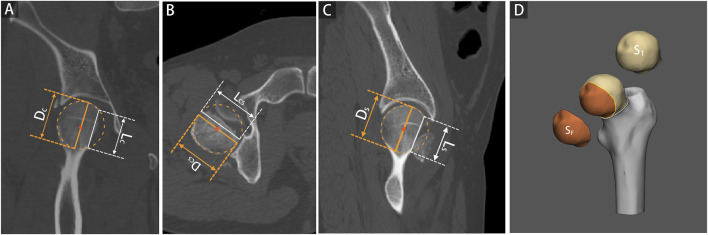
2D and 3D parameter measurements based on 2D and 3D injury models of femoral head fractures. The percentage of maximum defect length (PMDL) was measured as the ratio between the most significant residual defect length (L) and the optimum circle diameter for the femoral head (D), including coronal plane [Panel **(A)**, PMDL_C_ = L_C_/D_C_], cross-sectional [Panel **(B)**, PMDL_CS_ = L_CS_/D_CS_], and sagittal plane [Panel **(C)**, PMDL_S_ = L_S_/D_S_]. The percentage of fracture area (PFA) was determined as the ratio between the fracture area and the total area of the femoral head [Panel **(D)**, PFA = S_F_/S_T_].

Each 2D injury model of FHF, including coronal plane, cross-sectional, and sagittal plane, were separately used to measure the percentage of maximum defect length (PMDL). The 2D parameter in each 2D injury model was defined as the ratio between the most significant residual defect length and the optimum circle diameter of the femoral head (Wells et al., 2017; Hesper et al., 2018), (Figures 1A–C). Subsequently, the PMDL of each plane and average PMDL across three planes were determined. The 3D parameter of the percentage of fracture area (PFA) in each 3D injury model was defined as the ratio between the fracture area and total area of the femoral head (Figures 1D). Additional details of the measurement process of the 3D parameter are also given in the supplementary appendix.

To test the repeatability and reliability of PMDL and PFA parameters, 100 randomly selected FNFs were independently measured by two investigators using 2D-CT and 3D-CT images for inter-observer analysis. One of the investigators conducted two additional measurements 1 month apart for intra-observer analysis.

### Statistical analysis

Qualitative data were presented as a percentage whereas quantitative data were expressed as the mean with standard deviation (SD) using SPSS 24.0 software (IBM SPSS Inc., Armonk, New York). Reproducibility and agreement of parameters were tested using Bland-Altman (Bland and Altman, 1986) and intraclass correlation (ICC) (Rousson et al., 2002). The ICCs were interpreted according to a method by Landis and Koch (Landis and Koch, 1977). Factors significantly associated with FNF, including the injured side, age, gender, Pipkin classification, the 2D parameters, and the 3D parameter, were determined by binary logistic regression analysis. The diagnostic accuracy of significant 2D or 3D parameters was determined from the area under receiver operating characteristic curves or AUCs. Optimal cutoff value of each parameter was calculated using the receiver operating characteristic (ROC) curve. Patients were grouped according to the cutoff of the 2D parameter, with higher diagnostic accuracy, and the 3D parameter, respectively. Differences between groups were analyzed using the chi-square test or Fisher exact probability method for dichotomized values and the Mann-Whitney test for continuous values. Differences were considered statistically different at *p* < 0.05. The sensitivity, specificity, likelihood ratios, and positive and negative predictive values for different parameter cut-off values were also calculated.

Power calculation was performed by using PASS 15 Power Analysis and Sample Size Software (2017). NCSS, LLC. Kaysville, Utah, United States, ncss.com/software/pass. The primary outcome measures were cutoff values and area under the curve (AUC) of ROC curves for the FNF occurrence prediction. The secondary outcomes included the reproducibility and agreement of parameters and subgroup analyses based on cutoff values.

## Results

### Baseline characteristics

After screening 228 patients (228 hips) in our hospital’s orthopedic database, 19 patients were excluded. Among those excluded were eleven patients with insufficient or poor-quality CT images and two patients with an unclosed epiphyseal line of the femoral head. Five more patients were excluded for having a pathological fracture or skeletal immaturity. One patient with iatrogenic FNF was excluded for lacking CT images. Therefore, 209 hips in 209 patients comprising 102 left hip injuries (48.8%) and 107 right hip injuries (51.2%), were finally analyzed. Of these, 12 patients had FHF and FNF, including 7 Pipkin III, 2 Pipkin IV, 1 iatrogenic FNF during closed reduction, and 2 refractures of the femoral neck after internal fixation without trauma or fall.

### Parameter reproducibility and agreement test

**FIGURE 2:**
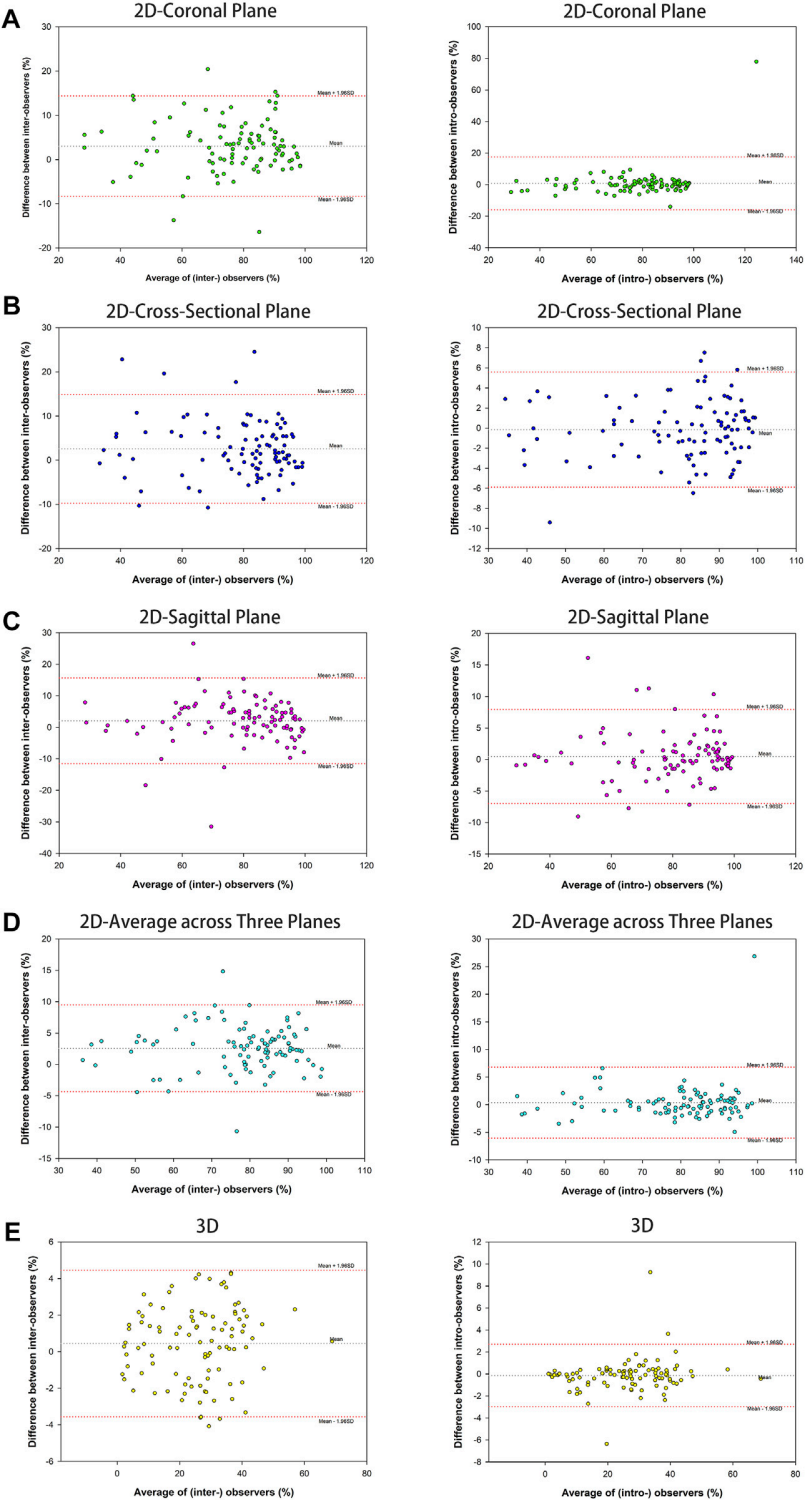
Bland-Altman plots of parameter measurement showed high assessment agreement of inter-observers and intra-observers, containing 2D parameter of the percentage of maximum defect length [coronal plane **(A)**, cross-sectional **(B)**, sagittal plane **(C)**, and average across three planes **(D)**] and 3D parameter of the percentage of fracture area **(E)**.

The results showed an almost perfect inter-and intra-observer reliability with ICC ≥0.887 (95% CI 0.837–0.922) for 2D parameters tested. Similarly, an almost perfect inter-and intra-observer reliability was found for the 3D parameter with ICC ≥0.987 (95% CI 0.981–0.992) (Table 2). The Bland–Altman analysis of 2D and 3D parameters measured by the two observers also showed high concordance (Figure 2).

**TABLE 2:** Inter-observer and intra-observer reliability.

Parameters		Inter-observer reliability		Intra-observer reliability	
		ICC	95% CI	ICC	95% CI
Two-dimensional Measurement^a^	Coronal plane	0.926	0.845–0.959	0.887	0.837–0.922
	Cross-sectional plane	0.926	0.873–0.954	0.986	0.979–0.990
	Sagittal plane	0.915	0.870–0.944	0.976	0.965–0.984
	Average	0.955	0.849–0.980	0.975	0.963–0.983
Three-dimensional Measurement^b^		0.987	0.981–0.992	0.994	0.991–0.996

ICC: intraclass correlation coefficient; CI: confidence interval.

^a^ Two-dimensional measurement of the percentage of maximum defect length.

^b^ Three-dimensional measurement of the percentage of fracture area.

### Correlation analysis between parameters and FNF occurrence

The binary logistic regression analysis of sex, age, injury side, Pipkin classification, 2D parameters, and 3D parameter showed that all 2D parameters except the sagittal plane and 3D parameter were significant predictors of FNF occurrence (*p* < 0.05) (Tables 3, 4).

**TABLE 3:** Relationship between femoral neck fracture and patient characteristics for the 209 patients with femoral head fracture by univariate binary logistic regression analysis.

Variable	Femoral head fracture with femoral neck fracture	
	OR (95% CI)	*p*-value
Side of injury	0.665 (0.204–2.168)	0.499
Age	1.011 (0.977–1.046)	0.528
Sex	1.441 (0.372–5.586)	0.597
Pipkin classification		
I	Reference	
II		1.000
III		0.999
IV		0.999
2D Measurement-Percentage of maximum defect length		
Coronal plane	1.212 (1.089–1.348)	<0.001
Cross-sectional plane	1.363 (1.152–1.611)	<0.001
Sagittal plane	1.256 (1.098–1.437)	0.001
Average	1.470 (1.229–1.757)	<0.001
3D Measurement-Percentage of fracture area	1.080 (1.023–1.140)	0.005

**TABLE 4:** Relationship between femoral neck fracture and patient characteristics for the 209 patients with femoral head fracture by multivariate binary logistic regression analysis.

Variable	Femoral head fracture with femoral neck fracture	
	OR (95% CI)	*p*-value
2D measurement-Coronal plane		
Side of injury	0.318 (0.023–4.331)	0.390
Age	1.022 (0.955–1.094)	0.524
Sex	1.232 (0.070–21.788)	0.887
Pipkin classification		
I	Reference	
II		1.000
III		0.999
IV		0.999
Percentage of maximum defect length	1.257 (1.009–1.566)	0.042
2D Measurement-Cross-sectional plane		
Side of injury	1.080 (0.037–31.721)	0.964
Age	1.049 (0.939–1.173)	0.399
Sex	4.610 (0.085–249.166)	0.453
Pipkin classification		
I	Reference	
II		1.000
III		0.999
IV		1.000
Percentage of maximum defect length	1.997 (1.121–3.557)	0.019
2D Measurement-Sagittal plane		
Side of injury	0.784 (0.053–11.491)	0.859
Age	1.044 (0.965–1.130)	0.286
Sex	0.718 (0.029–17.977)	0.840
Pipkin classification		
I	Reference	
II		1.000
III		0.998
IV		0.999
Percentage of maximum defect length	1.308 (0.969–1.766)	0.080
2D Measurement-Average		
Side of injury	0.843 (0.031–22.949)	0.920
Age	1.018 (0.925–1.121)	0.710
Sex	1.650 (0.009–298.304)	0.850
Pipkin classification		
I	Reference	
II		1.000
III		0.998
IV		1.000
Percentage of maximum defect length	1.789 (1.102–2.903)	0.019
3D Measurement		
Side of injury	0.149 (0.008–2.651)	0.195
Age	1.008 (0.930–1.093)	0.842
Sex	0.613 (0.032–11.628)	0.744
Pipkin classification		
I	Reference	
II		1.000
III		0.999
IV		1.000
Percentage of fracture area	1.254 (1.012–1.554)	0.038

### ROC analysis for FNF occurrence prediction

**FIGURE 3:**
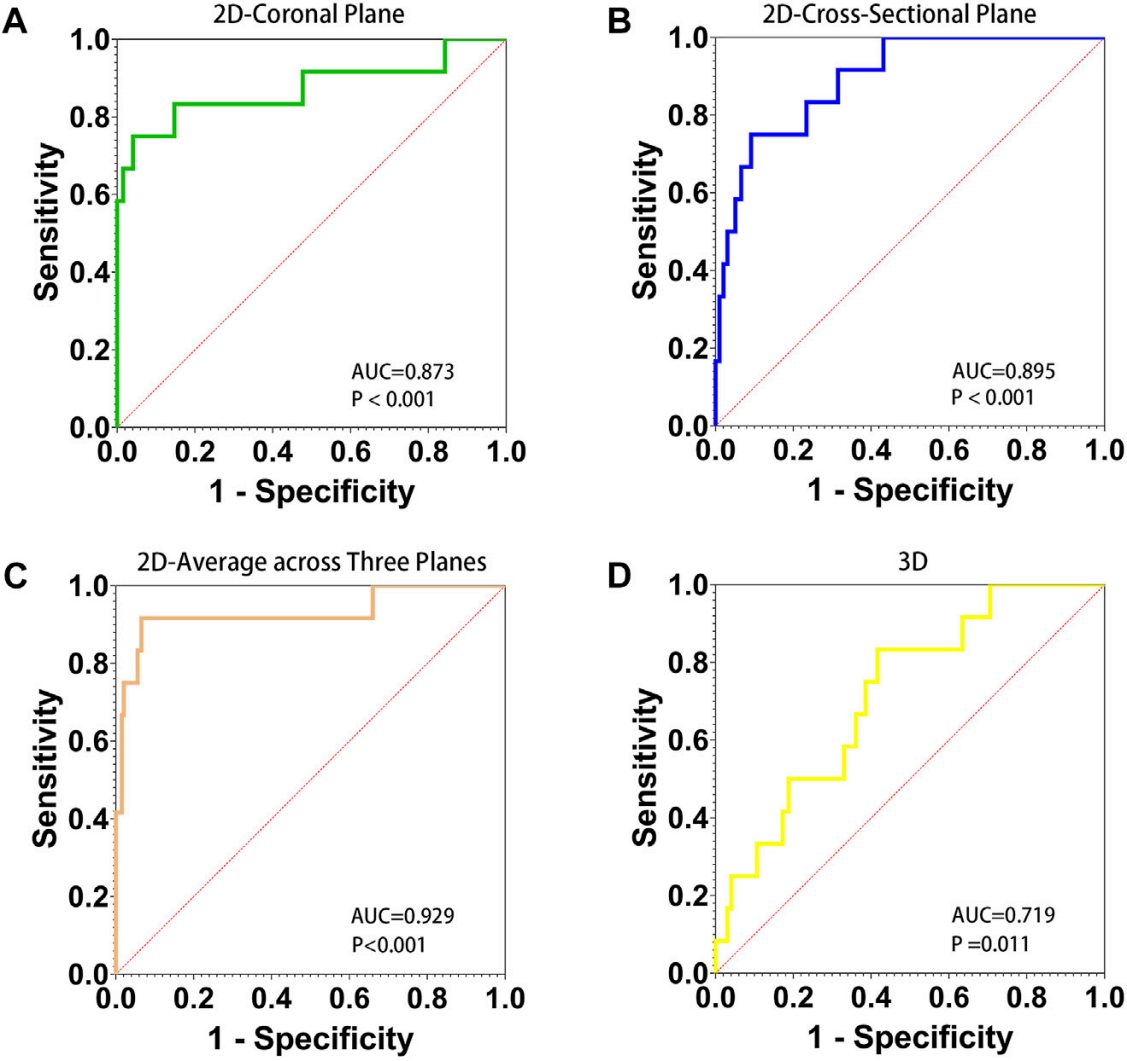
AUC, area under the ROC curve. Receiver operating characteristic (ROC) curve for 2D and 3D parameters to predict refracture of the femoral neck after femoral head fractures. The diagnostic accuracy of 2D parameter of the percentage of maximum defect length of coronal plane **(A)**, cross-sectional **(B)**, average across three planes **(C)**, and 3D parameter of the percentage of fracture area **(D)** was shown.

The AUC for the average PMDL three planes with a cutoff value of 91.65% was favorable and significantly better compared with that for 2D parameters (*p* < 0.001). The results of ROC analysis showed that the optimal cutoff value of the 3D parameter was 29.68% (Figure 3).

### Post-hoc power analysis

The *post hoc* power calculation based on AUCs in the primary outcome data showed that the 2D parameter, average PMDL across three planes, and the 3D parameter could predict FNF occurrence. The study power of 2D parameter and 3D parameter was 100% and 74.24%.

### Diagnostic accuracy analysis

**FIGURE 4:**
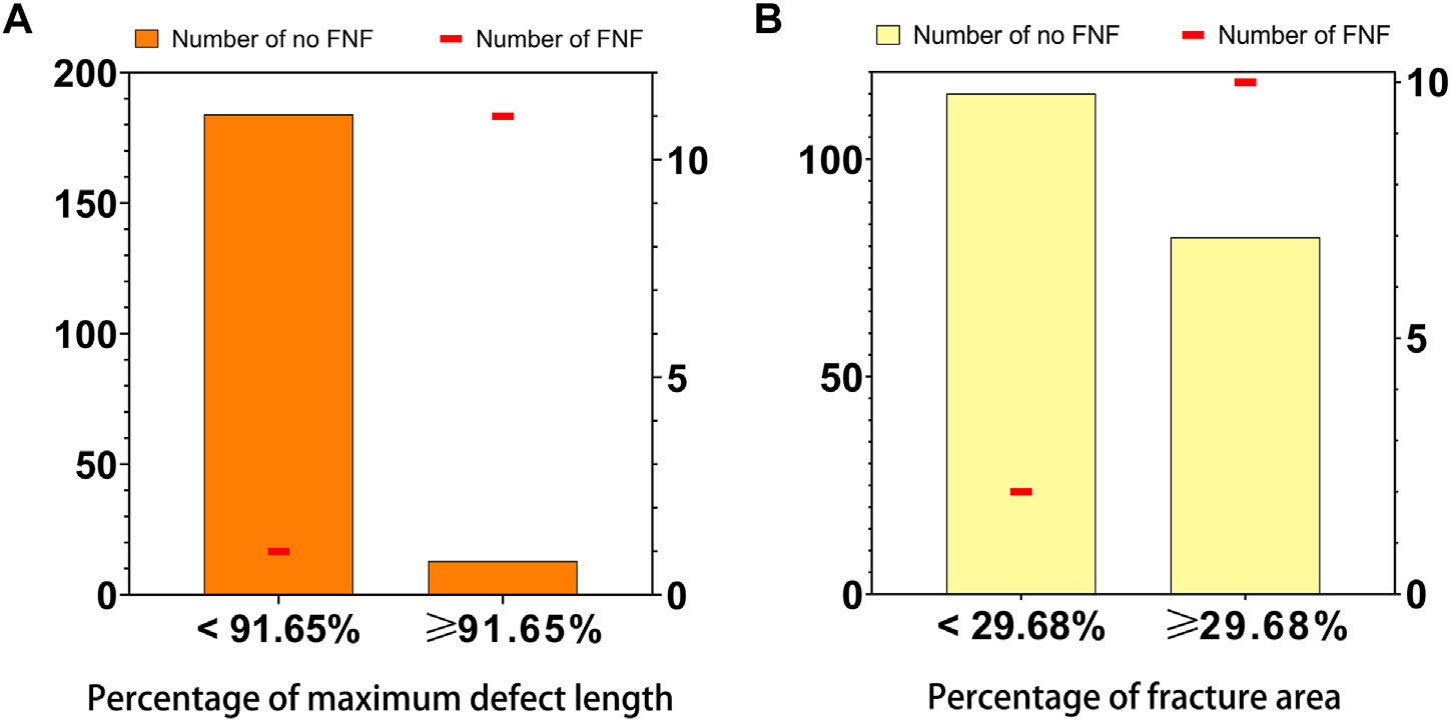
Distribution of 2D parameter **(A)**, the average percentage of maximum defect length across three planes, and 3D parameter **(B)**, the percentage of fracture area, and the number of the femoral neck fracture in the 209 patients with femoral head fractures. Numbers were indicated [femoral head fracture with (line in red) or without (bar in orange or yellow) femoral neck fracture] in the relevant group.

Subgroups were analyzed separately based on the cutoff values of 2D (average PMDL across all three planes) and 3D (PFA) parameters. Figure 4 shows the distribution of single FHFs and the number of FHFs combined with FNFs in the indicated subgroups. In total, 11/24 of FHFs with average PMDL ≥91.65% across all three planes were FNFs whereas 1/185 of FHFs with an average PMDL <91.65% across all three planes were FNFs (*p* < 0.001). In addition, 10/92 of FHFs with PFA ≥29.68% were FNFs whereas 2/117 of FHFs with PFA <29.68% were FNFs (*p* = 0.005) (Table 5).

**TABLE 5:** Data for 209 patients with femoral head fractures.

Variable	2D measurement of average across three planes			3D measurement		
	Percentage of maximum defect length	Percentage of maximum defect length	*p*-value	Percentage of fracture area	Percentage of fracture area	*p*-value
	<91.65%	≥91.65%		<29.68%	≥29.68%	
Number	185 (100)	24 (100)		117 (100)	92 (100)	
Side of injury			0.576^a^			0.100^a^
Left	89 (48.1)	13 (54.2)		63 (53.8)	39 (42.4)	
Right	96 (51.9)	11 (45.8)		54 (46.2)	53 (57.6)	
Age	38.0 (25.5–50.0)	37.5 (23.3–53.3)	0.873^b^	36.0 (24.5–50.0)	39.5 (26.0–54.0)	0.282^b^
Sex			0.581^c^			0.889^a^
Male	148 (80.0)	21 (87.5)		95 (81.2)	74 (80.4)	
Female	37 (20.0)	3 (12.5)		22 (18.8)	18 (19.6)	
Pipkin classification			<0.001^c^			0.006^c^
I	3 (1.6)	0 (0.0)		3 (2.6)	0 (0.0)	
II	53 (28.6)	7 (29.2)		26 (22.2)	34 (37.0)	
III	1 (0.5)	7 (29.2)		2 (1.7)	6 (6.5)	
IV	128 (69.2)	10 (41.7)		86 (73.5)	52 (56.5)	
Simplified Pipkin classification			0.912^a^			0.057^a^
I + II	56 (30.3)	7 (29.2)		29 (24.8)	34 (37.0)	
III + IV	129 (69.7)	17 (70.8)		88 (75.2)	58 (63.0)	
Femoral neck fracture	1 (0.5)	11 (45.8)	<0.001^c^	2 (1.7)	10 (10.9)	0.005^a^

^a^ Number of patients (percentage) and *p*-values determined with the chi-square test.

^b^ Median (interquartile range) and *p*-values derived with the Mann-Whitney test.

^c^ Number of patients (percentage) and *p*-values determined with the Fisher exact test.

The sensitivity and specificity of the 2D parameter were 91.7% and 93.4%, and the 3D parameter were 83.3% and 58.4%. The positive likelihood ratio and negative likelihood ratio of 2D parameter for FNF prediction were 13.8 and 0.09, and 3D ones were 2.0 and 0.29. The predictive positive value and negative predictive value for 2D parameter were 45.83% and 99.46%, and the 3D parameter were 10.87% and 98.29%.

## Discussion

The results of this study confirmed our hypothesis that the 2D and 3D CT-based parameters were reliable predictors of FNF in patients with FHFs. Furthermore, all parameters in 2D and 3D injury models were statistically significant when expressed as a continuous value. However, we presented 2D and 3D parameters for the injury degree of the femoral head as a dichotomized value. Therefore, knowledge of “50% FNF in patients with FHFs when average PMDL across all three planes exceeds 91.65% or PFA exceeds 29.68%” could be applied in clinical practice. To our knowledge, this is a single large consecutive case series of FHFs to measure 2D and 3D CT-based parameters and investigate their association with FNF in patients with FHFs. The newly exact measurement of injury degree of femoral head, based on 2D and 3D model analysis, has been established to assess the fracture risk of femoral neck, and it could be treated as a feasible adjunctive diagnostic tool in identifying FNFs in patients with FHFs. In this study, all parameters based on CT-based injury model was of great value in clinical application and research due to its convenience and favorable diagnostic performance.

In the past decade, a positive role of the position of the femoral head in relation to the acetabulum in iatrogenic FNF prevention was proposed by a small series of studies (Sy et al., 2005; Park et al., 2016). However, this conclusion remained controversial. Sy et al. (2005) reported that FNF might occur when femoral head defects were caught on acetabular rim during reduction movement. In addition, the femoral head remained attached above and posteriorly to the acetabulum and rotated less than 90° in four cases. In another retrospective study by Park et al. (2016), five of the nine patients experienced FNFs during attempted closed reduction. Fragments of the femoral head fracture were retained in the acetabulum in these series while the remaining component was posterior and superior relative to the acetabulum. In addition, the remaining component was engaged or locked against the sharp rear angle of the acetabulum. Therefore, the injury degree of femoral head is essentially a significant predictor of the incidence of FNF in FHFs during attempted reduction (Davis, 1950; Thompson and Epstein, 1951; Henle et al., 2017). However, because of poor methodological rigor inherent in qualitative reports, a relatively small sample size, and lack of measurement criterion and comparison group, the level of evidence for clinical application of positioning of the femoral head in relation to the acetabulum is very low.

**FIGURE 5:**
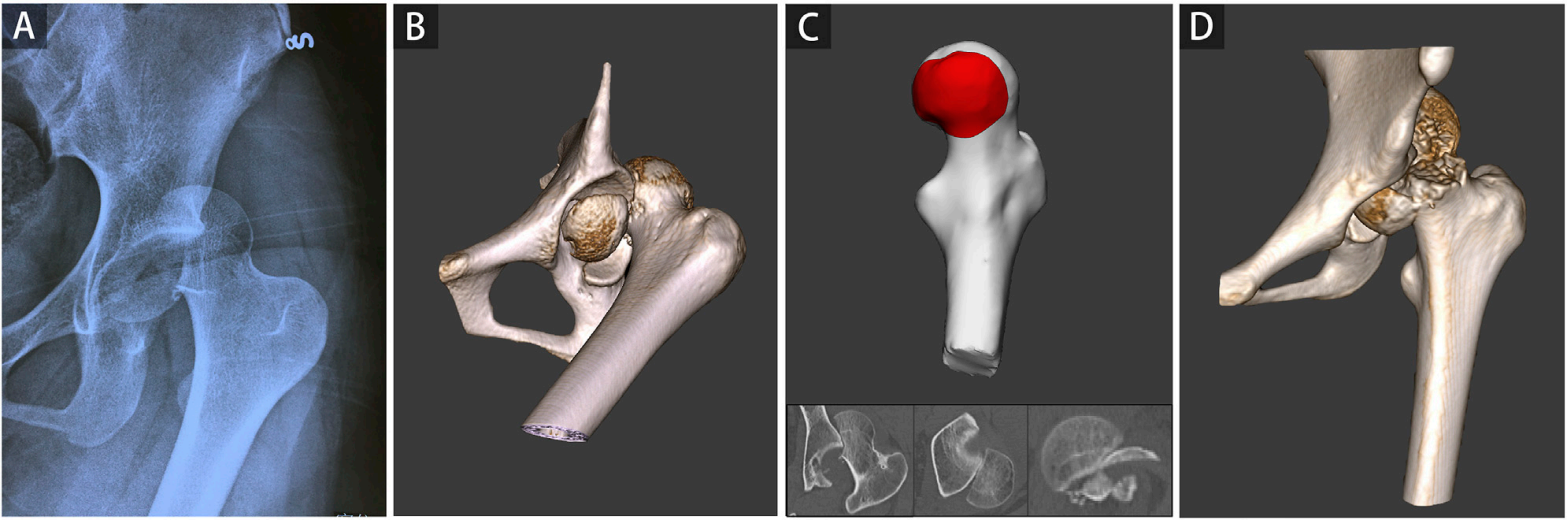
A representative case of iatrogenic femoral neck fracture during closed reduction was shown. A 16-year-old girl involved in a severe traffic accident was diagnosed with femoral head fracture based on X-ray **(A)** and CT **(B)**. The average percentage of maximum defect length across three planes of 92.80% in the 2D injury model and the percentage of fracture area of 38.33% in the 3D injury model were determined **(C)**. A refracture of the femoral neck occurred during closed reduction **(D)**.

**FIGURE 6:**
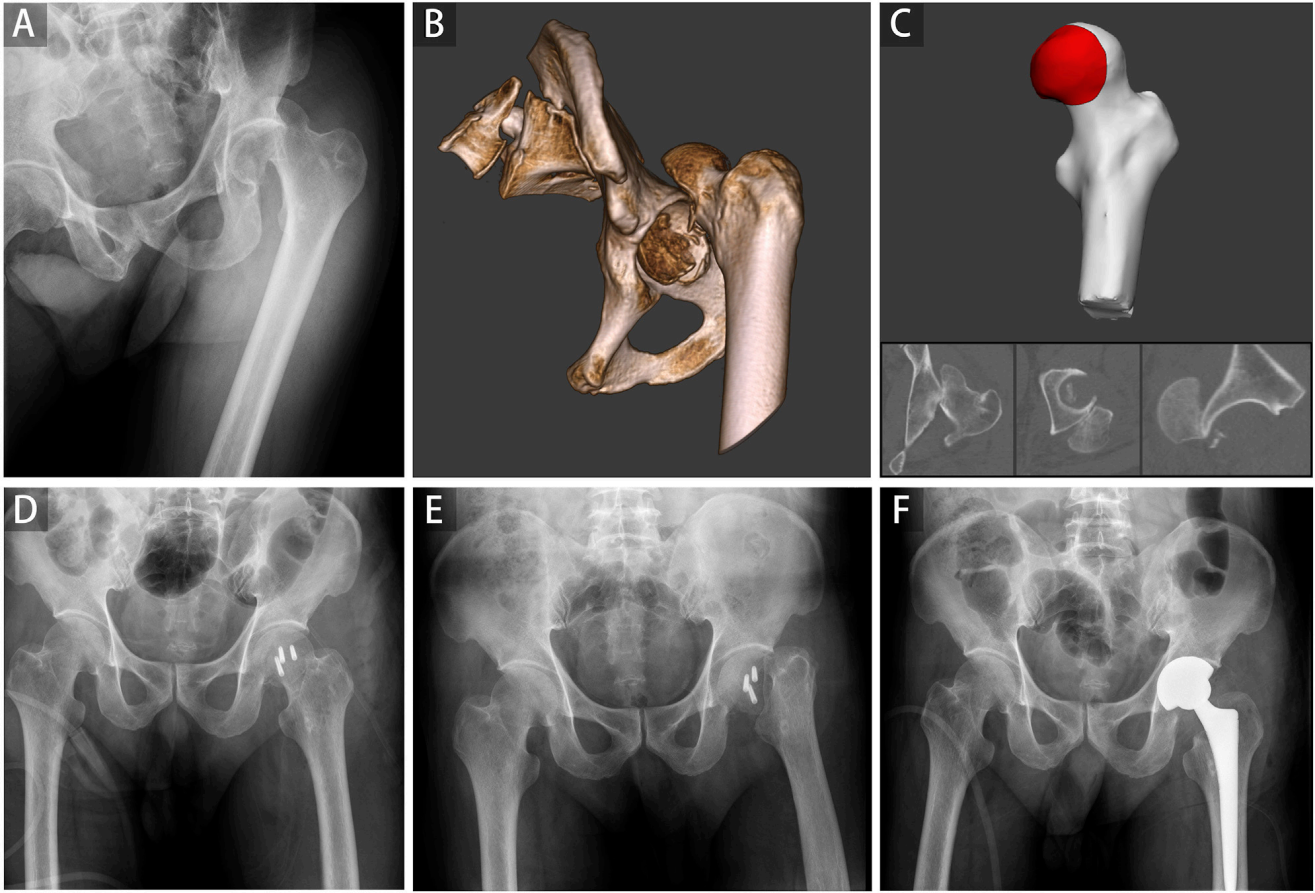
A representative case of the refracture of the femoral neck after internal fixation of the femoral head fracture was shown. A 62-year-old man involved in a severe traffic accident was diagnosed with femoral head fracture based on X-ray **(A)** and CT **(B)**. The average percentage of maximum defect length across three planes of 96.64% in the 2D injury model and the percentage of fracture area of 40.72% in the 3D injury model were determined **(C)**. Open reduction and internal fixation were conducted, and the postoperative radiograph showed good reduction status **(D)**. A refracture of the femoral neck occurred without trauma and fall 3 weeks postoperatively **(E)**. Finally, hip replacement surgery was performed **(F)**.

In the current study, 12 patients were found with FHF and FNF, including concomitant fracture (Pipkin III and IV), iatrogenic FNF during closed reduction (Figure 5), and femoral neck refracture after FHF internal fixation without trauma. All three kinds of FHFs with FNF were included in this study to make 2D and 3D parameters more universally applicable. The present case illustrates patient’s refracture of the femoral neck after FHF internal fixation without trauma or fall (Figure 6). No similar findings have been previously reported. Therefore, we speculated that a naturally favorable stress distribution mechanism could not be balanced by FHF internal fixation, which causes femoral neck refracture without trauma or fall. With approximately 70% of the articular surface of the femoral head engaging in load transfer, bone defects of the femoral head can cause significant load changes in the femoral head and neck after fracture (Greenwald and Haynes, 1972). Thus, ensuring anatomic congruity in the articular surface is important for effective management of FHFs (Beebe et al., 2016). However, due to the difficulty in restoring the natural anatomic hip structure, FHF combined with FNF is associated with a more dismal prognosis than a single FHF (Giannoudis et al., 2009). Hence, early identification of these patients could improve their prognosis by providing more aggressive treatment strategies. Our findings showed a high risk of FNF when the injury degree of the femoral head reached a critical value determined from 2D and 3D parameters.

This study also corroborates previous findings regarding the percentage of the femoral head fragments that can be removed in the treatment of FNFs (Epstein, 1974; Epstein et al., 1976). In one study, excellent clinical outcomes were reported for eight patients who had less than one-third of the femoral head removed (Epstein, 1974). In contrast, when more than one-third of the femoral head was removed, no complete functional recovery was due to too much stress in the hip joint (Epstein et al., 1976). Our results showed that patients with 3D parameter of PFA exceeding 30% were at high risk of developing FNF. Moreover, of the three 2D parameters (coronal plane, cross-sectional, and sagittal plane), the average PMDL across all three planes had the best diagnostic accuracy and measurement reliability. In addition, there was no standard position for patients to take the CT examination due to severe pain, and taking the average across three planes for each 2D parameter, combined with the 3D parameter based on 3D fracture reconstruction, could allowed for minimizing the impact on patients’ position and selective bias of each CT plane. Therefore, an integrated assessment of 2D-CT images would help improve the predictive performance of the parameters.

One patient with iatrogenic FNF was excluded from this study for lacking CT images. FHFs were rare but serious injuries caused by high-energy trauma (Alonso et al., 2000). A CT scan of the hips was needed for better diagnosis and treatment of the fractures (De Mauro et al., 2021). The CT scan helped understand features of FHF, including the femoral head fracture pattern, the congruity of the hip joint, and the presence or absence of intra-articular loose fragments, which may not be accurately detected in X-ray image (Ross and Gardner, 2012). Moreover, it is inappropriate to define the standard measurement condition using X-rays to predict FNF in patients with FHFs, due to overlapping bones, exposure differences, and different body positions. The feasibility of the 2D and 3D parameters was assured by routine CT examinations of this special injury type. On the other hand, multidimensional and comprehensive assessments of CT images ensured better predictive efficacy.

## Limitations

This study had some limitations. First, a retrospective design was adopted and the number of cases with a combination of FHF and FNF was small. However, the sample size of this type of fracture was larger compared with samples in previous studies, representing a strength of our study (Pipkin, 1957; Park et al., 2016; Scolaro et al., 2017; Keong et al., 2019). Moreover, a large sample of FHFs in consecutive series was taken, which helped minimize selection bias. In addition, the *post hoc* power calculation in this study demonstrated the 2D and 3D parameters had adequate power to predict FNF occurrence. Second, although the results demonstrated high repeatability and reliability of the two methods, no further comparison was made between 2D and 3D methods. However, using two distinct parameters, the 2D and 3D parameters with respective strengths and biases, could enable surgeons to lower estimation errors of the injury degree of the femoral head, thus further predicting the incidence of FNF in patients with FHFs with CT in clinical practice.

## Conclusion

In summary, the new measurement for injury degree of femoral head, based on 2D and 3D injury models with CT, appeared to be reliable to assess the fracture risk of femoral neck in patients with FHFs in the clinic practice. All new parameters, including average percentage of maximum defect length across all three planes in 2D parameter and percentage of fracture area in 3D parameter, indicated strong emergence of femoral neck fracture in patients with femoral head fractures. Thus, these parameters could be a feasible adjunctive diagnostic tool in identifying FNFs. In addition, this finding might also provide a theoretic basis for the investigation of the convenient digital-model in complex injurie analysis.

## Figures and Tables

**FIGURE A1:**
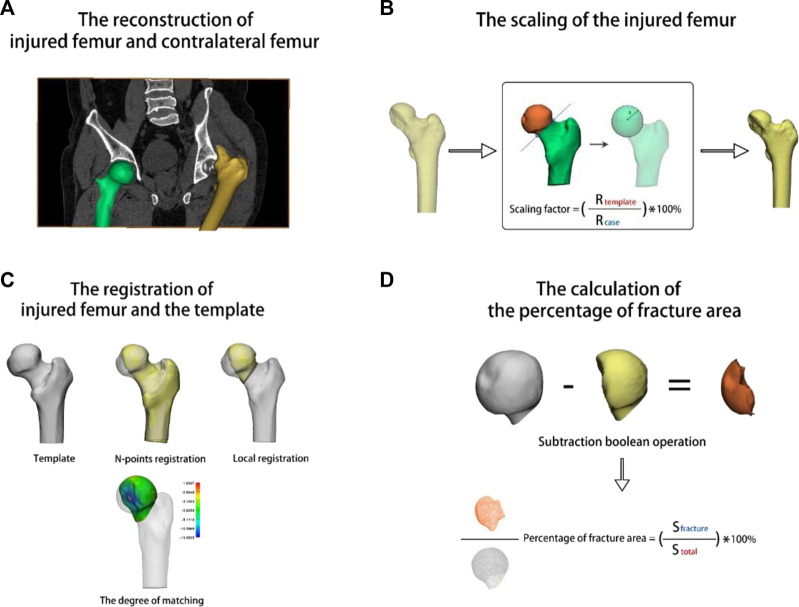
Flowchart of the 3D parameter measurement.

## Data Availability

The raw data supporting the conclusion of this article will be made available by the authors, without undue reservation.

## Appendix A:Details of 3D parameter measurement

Mimics software (version 20.0, materialise)

1. Reconstruct the 3D models of the proximal femur.
2. Export the 3-D model of the proximal femur of the injured (Figure A1, The proximal femur in yellow) to the 3-matic software.

3-matic (materialise)

1. Standardize the coronal, cross-sectional, and sagittal positions of the proximal femur of the injured to determine the femoral head part, according to the previous literature report [Reference: Wu S, Wang W, Zhang B, et al. A three-dimensional measurement based on CT for the posterior tilt with ideal inter-and intra-observer reliability in non-displaced femoral neck fractures. Comput Methods Biomech Biomed Engin. 2021; 24 (16): 1854–1861]. Then standardize the model size of the femoral head of the injured based on the scaling factor (Figure A1) to better match the femoral head of the template.
  1.1. Draw the caput sphere on the femoral head of the template and the injured, referred to the process in the above literature report, respectively. Then determine the radius of the sphere of the template and the injured, defined as *R*_*template*_ and *R*_*case*,_ respectively.
  1.2. Calculate the scaling factor, defined as the ratio between *R*_*template*_ and *R*_*case*_, used to adjust the model size of the femoral head of the injured to match the femoral head of the template better.
2. Achieve the best possible alignment between the femoral head of the template and the injured, referred to the process in the above literature report (Panel C, N-points registration and Local registration). The greener the color is, the higher the degree of matching is (Figure A1, The degree of matching).
3. Generate the fracture region through Subtraction Boolean Operation between the femoral head of the template and the femoral head of the injured (Panel D, Subtraction boolean operation).
4. Extract the area of the femoral head of the template defined as *S*_*total*_, then extract the area of the fractured region defined as *S*_*fracture*_ *.*
5. Calculate the percentage of fracture area, defined as the ratio between *S*_*fracture*_ and *S*_*total*_ (Figure A1).
